# Mitochondriogenesis and apoptosis: possible cause of vitamin A-mediated adipose loss in WNIN/Ob-obese rats

**DOI:** 10.1186/1743-7075-11-45

**Published:** 2014-09-25

**Authors:** Anamthathmakula Prashanth, Shanmugam M Jeyakumar, Lodhu Singotamu, Nemani Harishankar, Nappan V Giridharan, Ayyalasomayajula Vajreswari

**Affiliations:** Department of Lipid Biochemistry, National Institute of Nutrition (ICMR), Jamai Osmania, Hyderabad, 500 007 Andhra Pradesh India; Ultra Structure Unit, National Institute of Nutrition (ICMR), Jamai Osmania, Hyderabad, 500 007 Andhra Pradesh India; National Center for Laboratory Animal Sciences (ICMR), Jamai-Osmania, Hyderabad, 500 007 Andhra Pradesh India

**Keywords:** Vitamin A, Dietary supplementation, Uncoupling protein, Thermogenesis, Nuclear receptors, Adipose tissue, Apoptosis

## Abstract

**Background:**

Previously, we reported that vitamin A-enriched diet (129 mg/kg diet) intake reduces the adiposity development in obese rats of WNIN/Ob strain. Here, we hypothesize that dose lesser than 129 mg of vitamin A/kg diet would also be effective in ameliorating the development of obesity in these rats.

**Methods:**

Five-month-old male lean and obese rats designated as A & B were divided into four subgroups (I, II, III and IV) consisting of 8 rats from each phenotype and received diets containing 2.6 mg (control group), 26 mg, 52 mg and 129 mg vitamin A/kg diet as retinyl palmitate for 20 weeks. Body composition and morphological analysis of brown adipose tissue (BAT) was analyzed. Expression of uncoupling protein 1 (UCP1), retinoic acid receptor α (RARα) and retinoid X receptor α (RXRα) in BAT and levels of Bcl2 and Bax in epididymal white adipose tissue (eWAT) were determined by immunoblotting.

**Results:**

Vitamin A supplementation to obese rats at doses of 52 and 129 mg/kg diet showed reduced body weight gain and adiposity compared to control diet-fed obese rats receiving 2.6 mg of vitamin A/kg diet. In BAT of obese rats, vitamin A supplementation at doses of 26 and 52 mg of vitamin A/kg diet resulted in increased UCP1 expression with concomitant decrease in RARα and RXRα levels compared to control diet-fed obese rats. Further, transmission electron microscopy study revealed an increase in number of BAT mitochondria of obese rats supplemented with 26 and 52 mg of vitamin A/kg diet. Also, obese rats fed on 52 mg/kg diet resulted in increased apoptosis by altering the ratio of Bcl2 to Bax protein levels in eWAT. Notably, most of these changes were not observed in lean rats fed vitamin A-enriched diets.

**Conclusion:**

In conclusion, chronic consumption of 52 mg of vitamin A/kg diet seems to be an effective dose in ameliorating obesity possibly through mitochondriogenesis, UCP1-mediated thermogenesis in BAT and apoptosis in eWAT of obese rats. Therefore, the role of dietary vitamin A in correcting human obesity would be of unquestionable relevance and can only be addressed by future studies.

## Background

Obesity, an imbalanced energy metabolic disease is characterized by increased adipose tissue mass due to excessive energy accumulation in the form of triglycerides. Adipocytes accommodate the excess energy by its hyperplasic and/or hypertrophic mechanisms [1]. Though the regulation of both these process are not fully understood, adipocyte number is tightly regulated by pre-adipocyte recruitment, differentiation and by programmed cell death mechanism [2, 3].

Vitamin A has been shown to possess regulatory effects on energy balance and whole body adiposity [4, 5]. Previously, we reported that chronic feeding of diet rich in vitamin A (129 mg/kg diet) for a period of 2 months decreased body weight gain and adiposity in adult male obese rats of WNIN/Ob strain [4, 5]. This was majorly attributed to the activation of thermogenic pathway through up-regulation of the uncoupling protein 1(UCP1) gene [4]. As, the dose used in previous study (though within the safe upper limit) was substantially high, here we hypothesized that chronic feeding of vitamin A-enriched diets less than 129 mg/kg diet would be effective in ameliorating the development of obesity in these rats.

## Methods

### Animals, supplementation, and tissue collection

The WNIN/Ob mutant strain was developed from a 90-year-old Wistar [WNIN] inbred rat stock colony maintained at National Institute of Nutrition, India. WNIN/Ob, which arose spontaneously from WNIN in 1997 has homozygous lean (+/+) and obese (-/-) phenotypes. Rats of the obese phenotype are euglycemic and characterized by hyperphagia, hypertriglyceridemia, hypercholesterolemia, hyperleptinemia and hyperinsulinemia [4].

Male, five-month-old 32 lean and 32 obese rats of WNIN/Ob strain were obtained from the National Centre for Laboratory Animal Sciences (NCLAS) and broadly divided into two groups A and B respectively. Each group was further divided into four subgroups (AI, AII, AIII, AIV & BI, BII, BIII, BIV) consisting of 8 rats each. Subgroups AI and BI received 2.6 mg of vitamin A /kg diet and formed the control group. The stock diet consisted of 22.5% wheat flour, 60% Bengal-gram flour, 5% skimmed milk powder, 4% casein, 4% salt mixture and 0.5% vitamin mixture. The animals were provided with powdered chow of standard composition established at our institute containing all the recommended macro and micronutrients (56% carbohydrate, 18.5% protein, 8% fat, 12% fiber and adequate levels of minerals and vitamins) needed for rats. Subgroups AII and BII received 26 mg, subgroups AIII and BIII received 52 mg, while subgroups AIV and BIV received 129 mg of vitamin A /kg diet; as retinyl palmitate (a generous gift from Nicholas Piramal India Ltd.) respectively. All diets were identical with regard to all other ingredients except vitamin A content. Rats were housed individually with an ambient temperature 22.0 ± 1°C, relative humidity of 50-60%, 12-h:12-h light–dark cycle and fed their respective diets for a period of 20 weeks. At the end of experimental feeding, the animals were fasted in the evening for 12-h and the next day morning blood was collected from supra-orbital sinus via the inner canthus, and plasma samples were prepared. Later the animals were sacrificed by CO_2_ asphyxiation and various adipose tissues were excised in their entirety, weighed, frozen in liquid nitrogen, and stored at -80°C for further analysis. Animal experiment was approved by Institutional Animal Ethical Committee and conducted in accordance with the principle of the guide to the care and use of experimental animals.

### Body composition analysis by total body electrical conductivity

Two days prior to experimental sacrifice, total body composition was analyzed by small animal body composition analyzer (EM-SCAN, Model SA-3000 Multidetector, Springfield, USA) using Total Body Electrical Conductivity (TOBEC) method as described earlier [6]. This instrument measures total body electrical conductivity of small animals in a non-invasive manner. The rats were anaesthetized lightly with ether and placed in a carrier in the TOBEC chamber and 10 to 12 recordings were taken. The intra-assay coefficient of variation was less than 3.0%. In lean rats the estimation was carried out using coil with I.D. 3076 and in obese rats by coil with I.D. 3011. The following body composition parameters were obtained mathematically, where E stands for total electrical conductivity:

1. Lean body mass = 0.5E + (0.3 × total body weight)
2. Total body fat = total body weight ‒ lean body mass
3. Total body fat percent = (total body fat/ total body weight) × 100
4. Fat ‒ free mass = 16.28 + 0.4E

### Body temperature measurement

Body temperature was measured as rectal temperature by using BIO-pac MP-100 polygraph (Stoleting group of companies, CA, USA).

### Biochemical parameters

Serum and tissue retinol levels were determined by high-performance liquid chromatography as described previously [4]. Briefly, 100 μl of retinyl acetate (internal standard; 1 μg/mL) was added to 100 μl of serum and vortexed for 10–20 seconds. Hexane (120 μl) was added and contents were vigorously mixed but intermittently for 4 seconds, making sure that the bottom layer was thoroughly extracted. The tubes were centrifuged at 2200 rpm for 5 minutes and the upper hexane layer was carefully transferred to a fresh tube, evaporated under a stream of nitrogen and immediately reconstituted in methanol and injected for analysis by high-performance liquid chromatography (Shimadzu LC-6A model, Kyoto, Japan).

Liver and retroperitoneal white adipose tissue (WAT) were first washed free of blood, blotted and weighed. The tissues were then ground well with anhydrous sodium sulfate (thrice its weight) till a dry powder was obtained. About 100 mg of dry liver powder and 200 mg of dry retroperitoneal WAT powder was taken and extracted in 25 mL of diethyl ether and left overnight with occasional gentle shaking at 4°C. 500 μl of the ether extract was evaporated under a stream of nitrogen and reconstituted in methanol and injected for analysis by high-performance liquid chromatography.

### Transmission electron microscopy

Brown adipose tissue (BAT) was fixed by immersion in the Karnovsky fixative overnight at 4°C, dehydrated, cleared, and then resin-embedded. 600–900 A^0^ thick sections were obtained and stained with uranyl citrate and lead citrate (Taab laboratories, UK) and scanned using transmission electron microscope (Hitachi, H-7500, Japan) at 60–80 KV to assess morphology [7]. Photomicrographs were taken at 15000x magnification.

### DNA fragmentation analysis

Epididymal white adipose tissue (eWAT) DNA was isolated and analyzed for fragmentation as per the method of Gullicksen PS *et al.* [8]. 200 mg of tissue was homogenized in 1 mL of lysis buffer (10 mM Tris–HCl pH 8.0, 10 mM EDTA and 0.5% triton X-100) and centrifuged at 14,000 × g for 15 minutes at 4°C. The supernatant containing soluble (fragmented) DNA was transferred to a new tube. DNAzol (MRC, Cincinnati, USA) (0.5 ml) was added to the pellet containing insoluble (genomic) DNA. Both tubes were treated with RNase A (0.5 mg/ml) for 30 minutes at 37°C. Equal volumes of phenol/chloroform/isoamyl alcohol were added to the soluble DNA fraction, vortexed and centrifuged at 16,000 × g for 10 minutes at room temperature. The aqueous phase was collected and 5 μl of MgCl_2_ (<10 mM final conc.) and 1/2 vol of 7.5 M ammonium acetate were added and then mixed by inversion. The DNA was precipitated by addition of an equal volume of isopropyl alcohol. The samples were mixed and stored for 1 hour at -20°C. The DNA precipitate was pelleted by centrifugation at 10,000 × g for 30 minutes, and washed twice with 1 ml 70% ethanol followed by centrifugation at 10,000 × g for 3 minutes.

The genomic DNA fraction containing DNAzol was centrifuged at 16,000 × g for 3 minutes to sediment insoluble material. The supernatant was collected and DNA was precipitated by the addition of 1/2 vol of ethanol, mixed and stored for 3 minutes at room temperature. The DNA precipitate was sedimented by centrifugation at 10,000 × g for 5 minutes, and washed twice with 1 ml of 70% ethanol and centrifuged at 5,000 × g for 3 minutes. The DNA pellets from both fractions were air-dried and resolubilized in 25 μl Tris-EDTA buffer (pH 8.0). Soluble DNA (10 μl) and genomic DNA (5 μl) was loaded onto a 2% agarose gel, which was stained with ethidium bromide and visualized with UV light (DSS imagetech imager, India).

### Western blot analysis

BAT samples were homogenized in a buffer containing 250 mM sucrose, 10 mM HEPES pH 3.5, 0.5 mM EDTA, 0.1% BSA supplemented with 5% protease inhibitor cocktail and obtained various cellular fractions by differential centrifugation. Nuclear fraction was used to detect retinoic acid receptor α (RARα) and rexinoid receptor α (RXRα), using rabbit polyclonal antibodies (Santa Cruz Biotechnology, CA, USA), while uncoupling protein 1 (UCP1) protein levels were detected in mitochondrial fraction using polyclonal goat anti-UCP1 antibody (Santa Cruz Biotechnology, CA, USA). Immunoblotting of Bcl2 and Bax in eWAT was done as described previously [9] using polyclonal anti-bcl2 and anti-bax antibodies (Imgenex, CA, USA). Equal loading of the protein and transfer were ensured by staining the membrane with Ponceau S. Immuno-reactive proteins were detected by ECL advance western blotting detection kit (GE Healthcare, UK) and band density was analyzed by using quantity one software (GS-710 Imaging Densitometer- Bio-Rad, Hercules, CA, USA).

### Statistical analysis

Data are presented as mean ± SE. Differences among the various groups were assessed by one-way ANOVA; contrast between means was assessed by least-significance difference (LSD) post-hoc comparison. Statistical significance was determined at *P* < 0.05. The analyses were performed with SPSS 11.0 for windows (SPSS, Chicago, IL).

## Results

### Effects of vitamin A supplementation on biometric parameters

The effects of vitamin A supplementation on biometric parameters are shown in Table 1. There was a reduction in the body weight gain, total body fat and fat% without affecting food intake in vitamin A fed-obese rats (BIII & BIV respectively) receiving 52 and 129 mg/kg diet, while no such changes were observed in obese rats fed on 26 mg of vitamin A/kg diet (BII) as against the control diet-fed obese rats (BI). Obese rats receiving 52 mg of vitamin A/kg diet showed a significant reduction in both retroperitoneal white adipose tissue (rWAT) and eWAT, while the diet providing 129 mg of vitamin A/kg diet showed a reduction in rWAT alone. Further, the omental and brown adipose tissue weights remained unaltered upon feeding vitamin A-enriched diets (data not shown). Obese rats (BI) displayed lower rectal temperature than lean rats fed on control-diet (AI), which increased significantly upon vitamin A feeding. Except decreased fat%, no other changes in body composition, mass of various fat depots, food intake and rectal temperature were seen in identically-treated lean rats, as compared to control diet-fed lean rats (AI).

**Table 1:** **Effects of vitamin A supplementation on biometric parameters in WNIN/Ob rats**

Parameters	Lean				Obese			
	AI	AII	AIII	AIV	BI	BII	BIII	BIV
Initial body wt. (g)	352 ± 13.3^*a*^	374 ± 8.2^*a*^	351 ± 21.8^*a*^	362 ± 24.9^*a*^	628 ± 48.3^*b*^	594 ± 36.4^*b*^	574 ± 27.9^*b*^	591 ± 12.9^*b*^
Final body wt. (g)	424 ± 19.8^*a*^	455 ± 7.1^*a*^	441 ± 19.2^*a*^	454 ± 13.3^*a*^	926 ± 56.2^*b*^	883 ± 38.9^*bc*^	811 ± 33.4^*cd*^	778 ± 23.8^*d*^
Body wt. gain (g)	72 ± 18.9^*a*^	80 ± 10.1^*a*^	90 ± 25.1^*a*^	92 ± 15.9^*a*^	298 ± 22.1^*b*^	288 ± 10.9^*bc*^	237 ± 20.8^*cd*^	189 ± 17.2^*d*^
Food intake (g/day)	18.9 ± 0.57^*a*^	17 .4 ± 0.68^*a*^	19.4 ± 2.15^*a*^	18.8 ± 0.86^*a*^	27.9 ± 0.82^*b*^	29.3 ± 1.06^*b*^	28.1 ± 1.33^*b*^	26.8 ± 0.60^*b*^
Lean body mass (g)	361 ± 14.9^*a*^	398 ± 8.3^*a*^	389 ± 15.0^*a*^	402 ± 11.1^*a*^	410 ± 26.9^*a*^	399 ± 17.6^*a*^	376 ± 15.1^*a*^	362 ± 9.1^*a*^
Total body fat (g)	63 ± 5.6^*a*^	57 ± 3.2^*a*^	52 ± 5.6^*a*^	52 ± 4.8^*a*^	516 ± 30.8^*b*^	481 ± 15.5^*b*^	435 ± 18.5^*c*^	416 ± 15.3^*c*^
Fat%*	14.6 ± 0.75^*a*^	12.6 ± 0.77^*b*^	11.7 ± 0.90^*b*^	11.4 ± 0.84^*b*^	55.8 ± 0.69^*c*^	54.7 ± 0.40^*cd*^	53.7 ± 0.22^*d*^	53.4 ± 0.44^*d*^
rWAT ϕ	1.2 ± 0.07^*a*^	1.3 ± 0.23^*a*^	1.1 ± 0.19^*a*^	1.5 ± 0.19^*a*^	9.4 ± 1.02^*b*^	8.1 ± 0.19^*bd*^	7.6 ± 1.22^*cd*^	6.4 ± 0.45^*c*^
eWAT^**@**^	0.8 ± 0.22^*ac*^	0.7 ± 0.06^*ac*^	0.6 ± 0.05^*ac*^	0.7 ± 0.08^*ac*^	1.5 ± 0.26^*b*^	1.4 ± 0.35^*bd*^	0.9 ± 0.08^*cd*^	1.0 ± 0.12^*abc*^
Rectal temperature (°C)	37.6 ± 0.03^*ae*^	37.6 ± 0.31^*ae*^	37.6 ± 0.31^*ae*^	37.6 ± 0.22^*ae*^	36.0 ± 0.19^*b*^	37.7 ± 0.18^*a*^	38.3 ± 0.21^*cd*^	37.7 ± 0.12^*ce*^

Five-month-old male lean (A) and obese (B) rats were fed vitamin A at a dose of 2.6 (I), 26 (II), 52 (III) and 129 (IV) mg/kg diet for a period of 20 weeks. AI served as the control group for lean phenotype, while BI was control group for obese phenotype. Data are mean ± SE (n=8 for AI, AII, AIII, AIV; n=7 for BI, BII, BIV; n=6 for BIII). LBM, lean body mass; FAT%, fat percentage; eWAT, epididymal WAT; rWAT, retroperitoneal WAT.

*a–e* Mean values within a row not sharing a *common superscript* were significantly different by oneway ANOVA and LSD *post hoc* comparison (*P* < 0.05). *Total body fat expressed as a percentage of body weight. ϕMass of rWAT expressed as a percentage of body weight. **@**Mass of eWAT expressed as a percentage of body weight.

### Effect of vitamin A supplementation on retinol levels

Obese rats (BI) had higher serum retinol levels than their age and sex-matched lean counterparts fed on control diet (AI). Circulating retinol levels were not significantly affected by feeding vitamin A-enriched diets in both phenotypes. Total retinol levels in liver and rWAT were lower in obese control rats (BI) compared to their lean counterparts (AI). Significant increase in total retinol levels were seen in the liver (with all doses of vitamin A) and rWAT (with doses 52 and 129 mg of vitamin A/kg diet) of both lean and obese rats, when compared to their respective control diet-fed rats (Table 2).

**Table 2:** **Effects of vitamin A supplementation on retinol levels in WNIN/Ob rats**

Parameters	Lean				Obese			
	AI	AII	AIII	AIV	BI	BII	BIII	BIV
Serum retinol (μg/dL)	28 ± 1.4^*a*^	28 ± 1.3^*a*^	26 ± 2.0^*a*^	29 ± 3.4^*a*^	40 ± 1.6^*b*^	41 ± 2.5^*b*^	46 ± 3.9^*b*^	43 ± 5.7^*b*^
Liver retinol (μg/g tissue)	967 ± 386^*a*^	3795 ± 211^*bc*^	4556 ± 465^*bc*^	9842 ± 1151^*d*^	375 ± 60^*a*^	2840 ± 221^*b*^	5031 ± 239^*c*^	8133 ± 883^*d*^
rWAT retinol (μg/g tissue)	4 ± 1.2^*ac*^	14 ± 3.0^*ade*^	19 ± 1.5^*de*^	41 ± 7.7^*b*^	2 ± 0.3^*c*^	11 ± 0.9^*acd*^	24 ± 3.4^*e*^	48 ± 5.6^*b*^

Five-month-old male lean (A) and obese (B) rats were fed vitamin A at a dose of 2.6 (I), 26 (II), 52 (III) and 129 (IV) mg/kg diet for a period of 20 weeks. AI served as the control group for lean phenotype, while BI was control group for obese phenotype. Data are mean ± SE (n=8 for AI, AII, AIII, AIV; n=7 for BI, BII, BIV; n=6 for BIII). rWAT, retroperitoneal WAT.

*a–e* Mean values within a row not sharing a *common superscript* were significantly different by oneway.

ANOVA and LSD *post hoc* comparison (*P* < 0.05).

### Effect of vitamin A supplementation on BAT UCP1, RARα and RXRα expression

**Figure 1:**
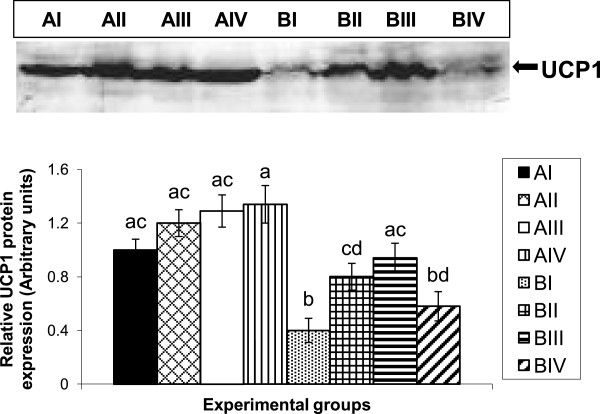
**Effect of vitamin A supplementation on BAT-UCP1 protein levels in lean and obese rats.** Five-month-old male lean **(A)** and obese **(B)** rats were fed vitamin A at a dose of 2.6 (I), 26 (II), 52 (III) and 129 (IV) mg/kg diet for a period of 20 weeks. Representative western blot showing the levels of UCP1 in BAT of lean and obese rats supplemented with various doses of vitamin A. Equal loading of the protein was ensured by staining the membranes with Ponceau S (image not shown). Bar graph shows the densitometric analysis of 3 rats, representing each of the dietary group. Bars are mean ± SE and are expressed relative to the mean value of lean control group (AI), which was set as 1. ^*a-d*^ Bars not sharing a *common superscript* are significantly different by one-way ANOVA and LSD *post hoc* comparison (*P* < 0.05).

Obese control rats (BI) showed an under-expression of BAT-UCP1 protein as compared to their age and sex-matched lean rats (AI). UCP1protein levels significantly increased after feeding vitamin A supplemented diets (26 and 52 mg/ kg diet) when compared with control diet-fed obese rats (BI); the effect was maximal (2.5 fold) at a dose of 52 mg/kg diet and diminished at 129 mg/kg diet. Though, UCP1 protein levels were increased by vitamin A supplementation in lean rats; they were not statistically significant (Figure 1).

**Figure 2:**
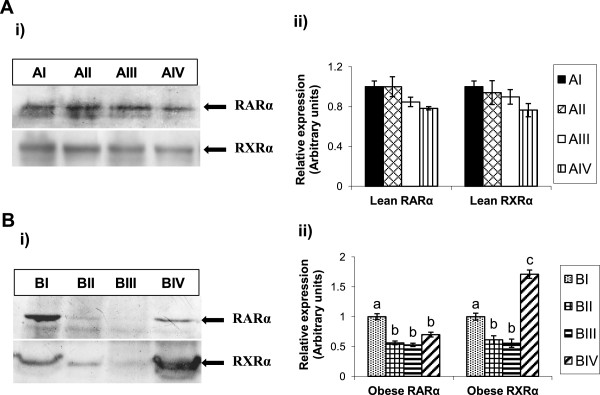
**Effect of vitamin A on BAT RARα and RXRα protein expressions in lean and obese rats of WNIN/Ob strain. A**. i) Representative western blot showing the levels of RARα and RXRα in BAT of lean rats & ii) Histogram represents the densitometric (arbitrary units) values of blot relative to control diet-fed lean rats (AI). **B**. i) Representative western blot showing the levels of RARα and RXRα in BAT of obese rats & ii) Histogram represents the densitometric (arbitrary units) values of blot relative to control diet-fed obese rats (BI). Equal loading of the protein was ensured by staining the membranes with Ponceau S (image not shown). Bars are given as means ± SE of 4 rats. Vitamin A-enriched diet was compared to stock diet of respective phenotypes. *P <* 0.05 was considered significant (one-way ANOVA). AI, BI- control diet, AII, BII- 2.6 mg/kg diet, AIII, BIII- 52 mg/kg diet & AIV, BIV- 129 mg/kg diet fed groups. ^*a-c*^ Bars not sharing a *common superscript* are significantly different by one-way ANOVA and LSD *post hoc* comparison (*P* < 0.05).

Basal expression of BAT nuclear receptors- RARα and RXRα protein levels were comparable between control diet-fed lean (AI) and obese rats (BI). Compared to control diet group (BI), vitamin A supplementation to obese rats decreased RARα levels at all doses. Interestingly, in obese rats RXRα expression decreased upon feeding with 26 and 52 mg of vitamin A/kg diet (BII and BIII respectively), while increased levels were observed with dose of 129 mg/kg diet (BIV) as compared to control diet- fed obese rats (BI). In contrast, no changes were observed in RARα and RXRα levels in lean rats fed vitamin A-enriched diets (Figure 2).

### Effect of vitamin A supplementation on BAT morphology

**Figure 3:**
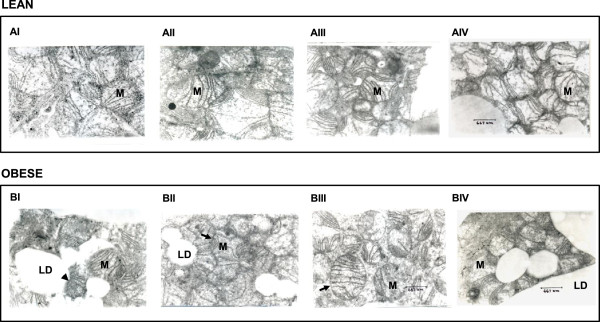
**Effect of vitamin A supplementation on BAT morphology.** Five-month-old male lean **(A)** and obese **(B)** rats were fed vitamin A at a dose of 2.6 (I), 26 (II), 52 (III) and 129 (IV) mg/kg diet for a period of 20 weeks. AI served as the control group for lean phenotype, while BI was the control group for obese phenotype. The BAT samples were fixed, and sections were stained with uranyl citrate and lead citrate for morphologic analysis. Photomicrographs were taken at 15000x magnification. *Scale bar*, 667 nm. The ruptured membranes observed in obese group (BI) are depicted by arrow heads. The intact mitochondria in BII and BIII are depicted by arrow. M- mitochondria, LD- lipid droplet.

We conducted a morphological study, wherein BAT sections of control and vitamin A-supplemented lean and obese rats were examined by electron microscopy for possible differences. Transmission electron microscopic (TEM) analysis of BAT revealed that control obese rats (BI) had relatively less number of mitochondria and also ruptured mitochondrial membranes as compared to their lean counterparts (AI) in a given field. Feeding of 26 and 52 mg of vitamin A/kg diet to obese rats (BII & BIII respectively) triggered an increase in the number of intact mitochondria, while no such changes were observed in obese rats (BIV) receiving 129 mg of vitamin A/kg diet as compared to control diet-fed obese rats (BI). Vitamin A supplementation (52 and 129 mg/kg diet) in lean rats also resulted in increased number of mitochondria as compared to the control diet-fed lean rats (AI) (Figure 3).

### Effect of vitamin A supplementation on Bcl2 and Bax expression in epididymal and retroperitoneal WAT

**Figure 4:**
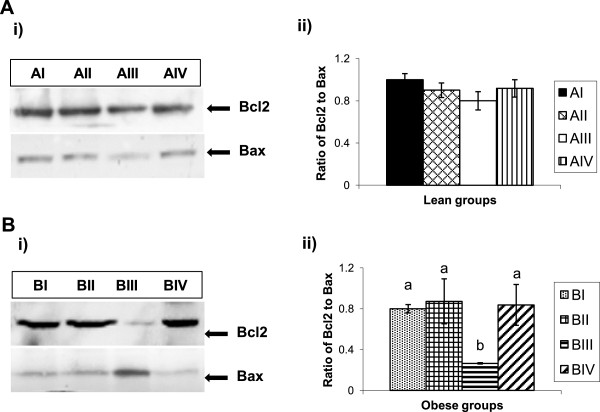
**Effect of vitamin A on epididymal WAT (eWAT) Bcl2 and Bax protein expressions in lean and obese rats of WNIN/Ob strain. A**. i) Representative western blot showing the levels of Bcl2 and Bax in eWAT of lean rats & ii) Histogram represents eWAT Bcl2–Bax ratio quantified densitometric values expressed relative to a value of 1 for control diet-fed lean rats (AI). **B**. i) Representative western blot showing the levels of Bcl2 and Bax in eWAT of obese rats & ii) Histogram represents eWAT Bcl2–Bax ratio quantified densitometric values expressed relative to a value of 1 for control diet-fed obese rats (BI). Equal loading of the protein was ensured by staining the membranes with Ponceau S (image not shown). Bars are given as means ± SE of 4 rats. Vitamin A-enriched diet was compared to stock diet of respective phenotypes. *P <* 0.05 was considered significant (one-way ANOVA). AI, BI- control diet, AII, BII- 2.6 mg/kg diet, AIII, BIII- 52 mg/kg diet & AIV, BIV- 129 mg/kg diet fed groups. ^*a-b*^Bars not sharing a *common superscript* are significantly different by one-way ANOVA and LSD *post hoc* comparison (*P* < 0.05).

Basal levels of Bcl2 and Bax were comparable in eWAT of lean (AI) and obese (BI) rats fed on control diet. Compared to control group (BI), Bcl2 and Bax levels significantly decreased and increased respectively in obese rats receiving 52 mg of vitamin A/kg diet (BIII) resulting in decreased ratio of Bcl2 to Bax, but not in other groups fed with either 26 (BII) or 129 mg of vitamin A/kg diet (BIV) (Figure 4). On the other hand, no changes in Bcl2 and Bax expression levels were observed in lean rats challenged with various doses of vitamin A (Figure 4). Vitamin A supplementation had no effect on Bcl2 and Bax expression levels in rWAT of both lean and obese rats (data not shown).

### Effect of vitamin A supplementation on DNA fragmentation in epididymal WAT

**Figure 5:**
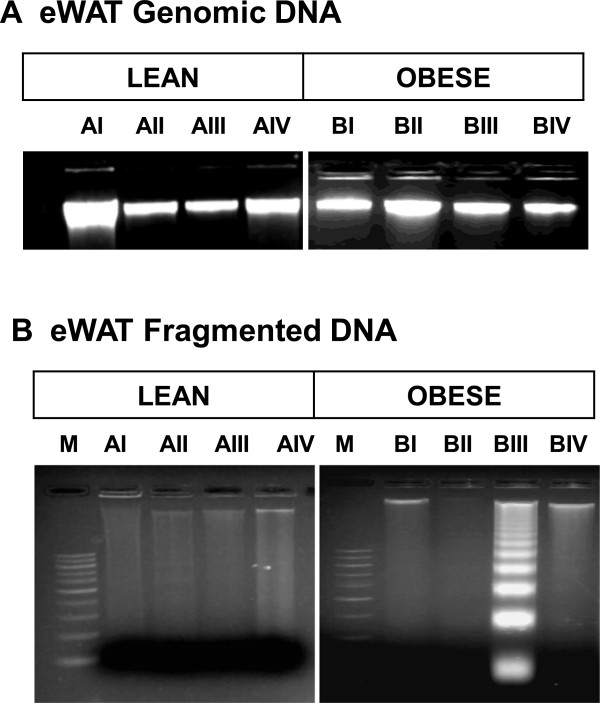
**Nucleosomal DNA fragmentation.** Representative picture of genomic DNA **(A)** and fragmented DNA **(B)** run on an agarose gel stained with ethidium bromide. M – DNA marker containing 100 bp ladder, AI and BI - lean and obese rats respectively fed on 2.6 mg of vitamin A/kg diet, AII and BII - lean and obese rats respectively fed on 26 mg of vitamin A/kg diet, AIII and BIII - lean and obese rats respectively fed on 52 mg of vitamin A/kg diet, AIV and BIV - lean and obese rats respectively fed on 129 mg of vitamin A/kg diet.

The decreased Bcl2 to Bax ratio in eWAT of obese rats fed 52 mg of vitamin A/kg diet was, further corroborated with the observed nucleosomal DNA fragmentation in this group. However, the same was not observed in obese rats (BII & BIV) fed with 26 and 129 mg of vitamin A/kg diet or in lean rats challenged with various doses of vitamin A (Figure 5).

## Discussion

We have shown previously that chronic feeding of vitamin A-enriched diet (129 mg/kg diet) to obese rats reduced body weight and adiposity without affecting food intake, possibly due to the activation of thermogenic pathway through up-regulation of the *BAT-UCP1* gene (4). In the present study, we observed that the consumption of vitamin A-enriched diet at doses of 52 and 129 mg/kg diet significantly decreased the gain in body weight, total body fat and retroperitoneal and epididymal WAT of obese rats as compared with their control diet-fed obese counterparts. It is interesting to note that the overall response of vitamin A supplementation was observed in obese but not in lean rats, suggesting possible differences in their vitamin A requirements/metabolism and the role of genetic-make up in eliciting such divergent response. In addition, vitamin A supplementation at any of the doses did not result in decreased food intake or other vitamin A toxicity symptoms. In order to elucidate the detailed mechanism involved in vitamin A-mediated weight reduction, we looked at the expression of thermogenic and apoptotic genes.

UCP1 is a brown-adipocyte-specific marker and the key molecular effector of thermogenesis [10]. Like other models of obesity (ob/ob, db/db mice and fa/fa rats), obese rats of WNIN/Ob strain show impaired BAT thermogenesis, which is associated with low UCP1 levels in BAT [11]. It is very well documented that vitamin A and its active metabolite; retinoic acid (RA) are positive regulators of UCP1. Feeding of vitamin A-deficient diets to rats and mice resulted in reduced BAT-UCP1 expression and increased adiposity, while all *trans*-RA treatment and vitamin A supplementation caused an increment of BAT thermogenic capacity and reduction of whole body adiposity, compared to their control animals [4, 12–15]. In line with these observations, our study also showed a marked induction of BAT-UCP1 expression in obese rats supplemented with 26 and 52 mg of vitamin A/kg diet. Increased thermogenesis results in elevated body temperature and, indeed, increased rectal temperature was observed in obese rats supplemented with vitamin A.

The responsiveness of UCP1 to retinoids is majorly mediated by nuclear transcription factors RARα and RXRα [15, 16]. Similar to earlier studies reported in both cultured primary brown adipocytes and BAT of mice, our observations also supported the down-regulatory effect of vitamin A in obese rats fed 26 and 52 mg of vitamin A/kg diet on RARα and, especially RXRα protein levels that paralleled the induction of UCP1, suggesting auto-regulated feed-back inhibition of the retinoids on the thermogenic system [15, 16]. Although, vitamin A is considered as a positive regulator of UCP1 gene expression, a recent study comparing the dose dependent activation of UCP1 in various mouse adipocytes revealed that intermediate concentrations of RA enhance UCP1 expression whereas higher concentrations failed to do so and this effect was mediated by the action of RARs [17]. Similarly, in the present study we observed the absence of UCP1 activation in BAT of obese rats at the highest dose (129 mg of vitamin A/kg diet) which could be due to increased RXRα levels and may be seen as turning-on of inhibitory mechanisms by vitamin A to prevent thermogenic activation. On the other hand, lean rats displayed no significant transcriptional activation of BAT-UCP1 in response to vitamin A-enriched diet, probably, as lean rats already had a maximal basal expression of UCP1.

For the first time, we report that TEM analysis of BAT reveals low number of mitochondria with disrupted membranes in these obese rats, which clearly suggest that not only the mitochondrial number but also the membrane structure is affected by obesity leading to augmented energy deposition as fat. Vitamin A is known to facilitate stable organization of well-developed lamellar cristae of the inner mitochondrial membrane, which is the hallmark of active BAT mitochondria [18]. Vitamin A-induced BAT-UCP1 expression in obese rats was also accompanied by morphological changes, notably, increased number of mitochondria in BAT and this could be conducive for the respiratory activity of the mitochondria and subsequent induction of thermogenic response. Thermogenic activation of BAT by vitamin A, is of practical relevance in the context of recent findings that active BAT depots are found in humans and cold-induced BAT activity is impaired in overweight healthy subjects [19]. Therefore, understanding the pathways that mediate the effect of vitamin A on UCP1 regulation could be essential for the design and implementation of pharmacological strategies to modulate thermogenic activation and increased energy expenditure.

In our study, we did not find any changes in the plasma retinol levels in response to supplementation with varied vitamin A contents of the diet. Furthermore, the data on liver retinol contents of lean and obese phenotypes suggest that hepatic storage of retinol could be the regulatory mechanism involved in plasma vitamin A homeostasis. Liver contains 66-75% of the body’s total retinol and is the major organ involved in the retinol storage and homeostasis. Next to liver, adipose tissue accounts for 15-20% of the total body retinoid stores and plays an active role in retinoid homeostasis and metabolism. Our observation, with regard to low liver and retroperitoneal WAT retinol stores, despite higher food intake, is suggestive of low uptake by these tissues or defective intestinal absorption of retinol in obese rats, as compared to lean rats. However, feeding various doses of vitamin A to obese rats raised hepatic and retroperitoneal WAT retinol levels comparable to those observed in lean rats on identical dietary regimen.

Adipose tissue mass is determined by the volume and/or the number of adipocytes, the former being regulated by lipolytic process and the latter by pre-adipocyte recruitment, differentiation and adipocyte apoptosis [20]. Various studies have substantiated the concept that adipocyte deletion by apoptosis contributes to ablation of adipose tissue and its loss during weight reduction [21–23]. All-*trans*-RA is known to induce differentiation and subsequent apoptosis in a variety of cell lines by modulating Bcl2 and Bax expressions. Bcl2 and Bax are prominent regulators of apoptosis, the former helps in prolonging cell survival and the latter antagonizes the function of Bcl2 and enhances the cellular susceptibility to apoptosis [24, 25]. Thus, the ratio of Bcl2 to Bax is deemed important in determining cell survival or death. The present study also demonstrates that vitamin A supplementation at a dose of 52 mg/kg diet induced apoptosis in the epididymal WAT of obese rats by decreasing Bcl2 and enhancing Bax expressions leading to decreased Bcl2 to Bax ratio and increasing nucleosomal DNA fragmentation, indicating that the decreased epididymal WAT in these rats is due to increased apoptosis.

It is interesting to note that obese rats fed on 26 mg of vitamin A/ kg diet had unaltered total body fat (despite increased UCP1 expression), which could be probably due to marginally increased food intake in this group. Surprisingly, high vitamin A treatment of obese rats had no effect on any of the parameters analyzed (BAT-mitochondriogenesis and UCP1 expression, epididymal WAT Bcl2 and Bax expressions). This rules out the possible role of vitamin A-mediated thermogenesis and apoptosis in lowering adiposity in obese rats fed 129 mg of vitamin A/kg diet, thereby implicating the role of preadipocyte recruitment and differentiation. In fact, lipoprotein lipase (LPL) and glycerol 3-phosphate dehydrogenase (GPDH) activities, markers of adipocyte differentiation, were found to be markedly elevated in control obese rats as compared with their lean counterparts. Further, high vitamin A supplementation (129 mg/kg diet) resulted in significant decrease in LPL and GPDH activities in obese rats, but not in the lean phenotype (data not shown). Therefore, the observed decrease in the retroperitoneal adipose tissue mass, adiposity and body weight in obese rats supplemented with 129 mg of vitamin A/kg diet could be attributed to inhibition of lipogenesis, due to decreased LPL and GPDH activities.

## Conclusions

The present study has clearly established the role of various doses of vitamin A in regulating obesity, using WNIN/Ob obese rats. Vitamin A supplementation at the dose of 52 mg/kg diet proved to be effective in bringing about a reduction in body weight and adiposity in obese rats by enhancing BAT thermogenic activity and inducing apoptosis in epididymal WAT. More importantly, increased understanding of the mechanisms and effectors involved in body weight regulation and their modulation by nutrients can contribute to novel pharmacological/dietary interventions for prevention and treatment of obesity.

## Abbreviations

UCP: Uncoupling protein
BAT: Brown adipose tissue
RAR: Retinoic acid receptor
RXR: Rexinoid X receptor
eWAT: Epididymal white adipose tissue
rWAT: Retroperitoneal white adipose tissue
TEM: Transmission electron microscopy.
